# Supplementary figures and images for: Temporal shifts in dengue epidemic in Guangdong Province before and during the COVID-19 pandemic: a Bayesian model study from 2012 to 2022
Source: PLoS Negl Trop Dis. 2025 Feb 3;19(2):e0012832. doi: 10.1371/journal.pntd.0012832 (PMC11805405; doi:10.1371/journal.pntd.0012832)

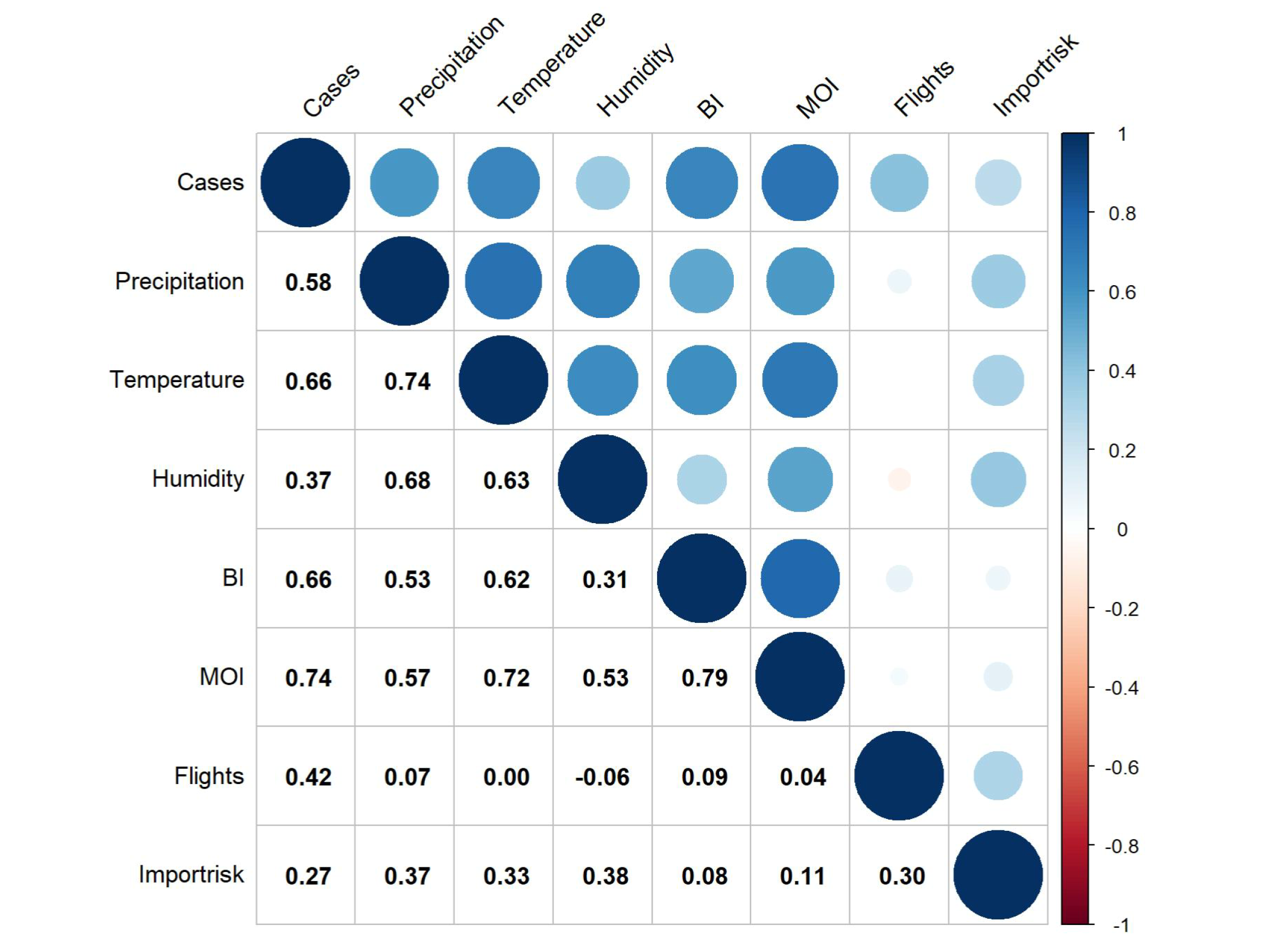

Supplement: S1 Fig — (TIF) [file pntd.0012832.s001.tif]

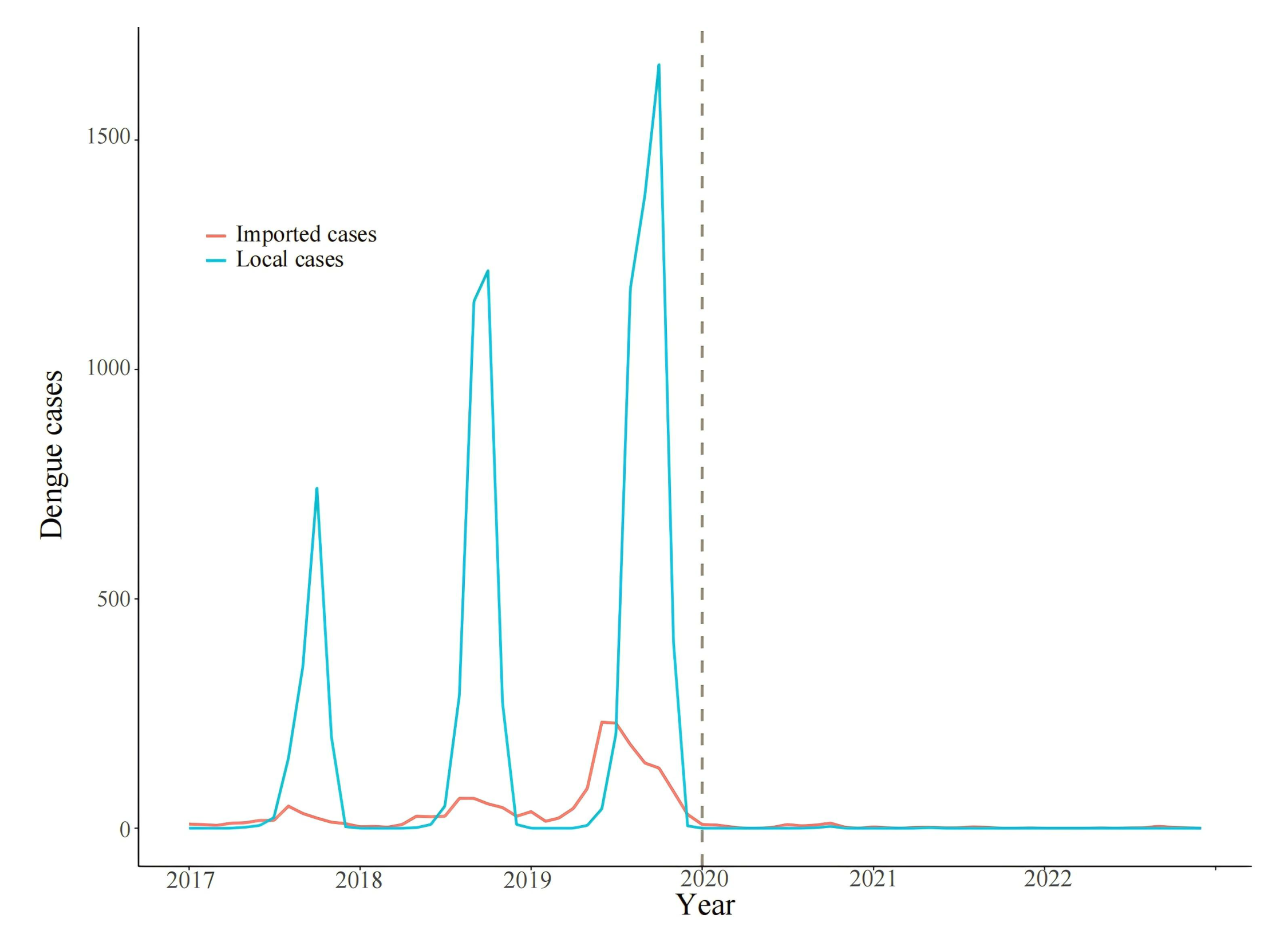

Supplement: S2 Fig — (TIF) [file pntd.0012832.s002.tif]
