# Supplementary material for: Temporal shifts in dengue epidemic in Guangdong Province before and during the COVID-19 pandemic: a Bayesian model study from 2012 to 2022
Source: PLoS Negl Trop Dis. 2025 Feb 3;19(2):e0012832. doi: 10.1371/journal.pntd.0012832 (PMC11805405; doi:10.1371/journal.pntd.0012832)
Supplement: S1 Table — (DOCX) [file pntd.0012832.s003.docx]

**S1 Table. BSTS model fitness and predictive effectiveness assessment.**

| **Model** | | **Model Fitness^a^** | | **Model prediction^b^** | |
| --- | --- | --- | --- | --- | --- |
| BSTS model | | R^2^ | MAPE | R^2^ | MAPE |
| $Model1$ | $Log[E(\mu_{t})]=Basic model+{Humid}_{t-e}+BI_{t-f}$ | 0.89 | 26.64% | 0.84 | 29.31% |
| $Model2$ | $Log[E(\mu_{t})]=Basic model+{Humid}_{t-e}+MOI_{t-f}$ | 0.85 | 28.24% | 0.86 | 29.55% |
| $Model3$ | $Log[E(\mu_{t})]=Basic model+BI_{t-f}$ | 0.89 | 26.92% | 0.84 | 28.82% |
| $Model4$ | $Log[E(\mu_{t})]=Basic model+MOI_{t-f}$ | 0.85 | 27.76% | 0.86 | 29.69% |

^a^ The time of Model fitness was 2012-2017; ^b^ The time of Model internal predictions was 2018-2019.

$$Basic model=\alpha+ {Temp}_{t-c}, df )+s({Precipitation}_{t-d}, df )+s( {Vect}_{t-f}, df )+s( {Imp}_{t-g}, df )+s( Time, df )+month+offset ( Pop )$$
