# Supplementary material for: Temporal shifts in dengue epidemic in Guangdong Province before and during the COVID-19 pandemic: a Bayesian model study from 2012 to 2022
Source: PLoS Negl Trop Dis. 2025 Feb 3;19(2):e0012832. doi: 10.1371/journal.pntd.0012832 (PMC11805405; doi:10.1371/journal.pntd.0012832)
Supplement: S2 Table — (DOCX) [file pntd.0012832.s004.docx]

**S2 Table. Sensitivity analysis for BSTS model estimation.**

| **Model** | | **Study period** | **Predicted cases** |
| --- | --- | --- | --- |
| Model 1 | $Log[E(\mu_{t})]=Basic model+{Humid}_{t-e}+BI_{t-f}$ | 2020-2022 | 6563 |
| Model 2 | $Log[E(\mu_{t})]=Basic model+{Humigd}_{t-e}+MOI_{t-f}$ | 2020-2022 | 6341 |
| Model 3 | $Log[E(\mu_{t})]=Basic model+BI_{t-f}$ | 2020-2022 | 7366 |
| Model 4 | $Log[E(\mu_{t})]=Basic model+MOI_{t-f}$ | 2020-2022 | 6777 |
