# Supplementary material for: Temporal shifts in dengue epidemic in Guangdong Province before and during the COVID-19 pandemic: a Bayesian model study from 2012 to 2022
Source: PLoS Negl Trop Dis. 2025 Feb 3;19(2):e0012832. doi: 10.1371/journal.pntd.0012832 (PMC11805405; doi:10.1371/journal.pntd.0012832)
Supplement: Attachment — Submitted filename: Response to Reviewers.docx [file pntd.0012832.s007.docx]

**Response to editors’ and reviewers’ comments**

**1. Section Editor**

**From SE:** Please address both reviewer's comments point by point, but issues raised by reviewer #2 about reframing the objectives of the study should be prioritized. I also agree that underreporting of dengue cases during the COVID pandemic must be consider.  The role of human movement of dengue virus is very important in dengue virus transmission dynamics, but I would argue that it is social movement between homes that are infested with vectors that may have been affected (family, friends, social contact).  It is very important to describe what control measures (vector control) are normally carried out for dengue control and if they were impacted during the pandemic.

**Response:**

Thank you for your insightful comments and suggestions. We have carefully considered each point and have made the following revisions to our manuscript:

1. For the objectives of the study, we have revised the research objectives to more accurately to reflect the scope and contribution of our study, with objectives updated as follows:

“We aimed to analyze the epidemiological characteristics of dengue in Guangdong Province, China, and to investigate the temporal shifts in dengue epidemic in Guangdong Province during the COVID-19 pandemic.”

2. Thank you for concerning the issue of potential underreporting of dengue cases. During the COVID-19 pandemic, the likelihood of dengue underreporting in Guangdong Province is minimal. The reasons are as follows:

**Firstly**, from January 2020 to November 2022, China implemented a 'dynamic zero-COVID' strategy, which effectively controlled the COVID-19 outbreaks. The incidence of COVID-19 in Guangdong was 12.6/100,000 population (15,989 cases) from January 2020 to June 2022 [1]. During this period, the healthcare system in Guangdong Province continued to function normally, allowing dengue cases to be treated promptly and thus reducing the likelihood of underreporting cases of dengue. **Secondly**, during 2020-2022, Guangdong implemented stricter health surveillance for fever symptoms, which are common in both COVID-19 and dengue. A person with fever symptom was promptly treated, this enhanced surveillance likely heightened the detection of dengue cases. Hospitals maintained sufficient sensitivity in diagnosing dengue cases, such as conducting NS1 antigen testing for suspected dengue virus cases in secondary and higher-level medical institutions across the province. Contrary to surveillance sensitivity before 2020, the monitoring and diagnosis of dengue cases have been enhanced during the COVID-19 pandemic. **Thirdly**, regarding dengue case reports, China classifies dengue as a Category B notifiable disease, requiring reporting within 24 hours of diagnosis. During the COVID-19 pandemic, Guangdong has consistently organized annual surveys and evaluations of the quality and management of notifiable infectious disease reporting across all levels of medical and disease control institutions in every city. The surveys indicated an average infectious disease reporting rate of 95%. Notably, the port cities of Guangzhou, Shenzhen, and Zhuhai have achieved reporting rates exceeding 99%, suggesting a low likelihood of underreporting for dengue. Nevertheless, there is still the possibility of underreporting, and we have added relevant descriptions in the limitations:

“First, some dengue cases may have been under-diagnosed and under-reported to some extent from 2020 to 2022, due to the burden of COVID-19 control and prevention.”

3. We fully acknowledge your perspective that implementing control measures is important for the control of dengue. According to your suggestions and the reviewers’ comments, we have added the relevant mosquito vector control measures in the Materials and Methods section (Line 155-173), as detailed below:

“As a high-risk region of dengue epidemic, Guangdong Province continued to pay attention to and even strengthen dengue prevention and control during the 2020-2022 period of COVID-19 pandemic, particularly during the dengue epidemic season. For instance, to prevent the risk of potential dengue importation, Guangdong implemented stricter health surveillance and screening measures for inbound travelers from regions where dengue was endemic, such as enhanced screening for Dengue Virus NS1 antigen. Regular monitoring of mosquito vector density is conducted to identify high-risk areas for vector breeding, facilitating timely vector control and breeding site sanitation. Based on the Joint Prevention and Control Mechanisms against on COVID-19 [2], Guangdong has strengthened collaboration among multiple departments, including the centers for disease control and prevention, hospitals, police, transportation, and urban management. If new local dengue cases are identified, these mechanisms ensure that the prevention and control responsibilities are firmly placed on local government, organizations, departments, and individuals. This has led to early case detection and vector control measures, facilitating the immediate control of local dengue outbreaks. Concurrently, health education was intensified to elevate public awareness of dengue prevention, and encourage the public to participate in the patriotic health action focusing on vector control and mosquito elimination, such as the removal of stagnant water and the reduction of mosquito breeding sites.”

**Reference:**

1.	Huang Z, Lin L, Li X, Rong Z, Hu J, Zhao J, et al. Evolution of COVID-19 dynamics in Guangdong Province, China: an endemic-epidemic modeling study. Archives of public health. 2024;82(1):173.

2.	The Joint Prevention and Control Mechanism of the State Council. [cited 2024 09 December]; Available from: <http://www.china.org.cn/english/china_key_words/2020-03/13/content_75810658.htm>.

As for Reviewer 1's request for additional time series model, please respond to the request but I would not require that for publication, again focus on the interpretation and implications of your findings and reconsider what are not very practical public health recommendations (see  comments by Review #2).  I agree that one must consider the impact of COVID on vectors.

**Response:**

Thank you for your valuable feedback. We have carefully thought about and responded to your suggestions in detail:

1. Following your suggestions, we have conducted additional analyses of ARIMA model and GAM model study. We subsequently compared the result from the ARIMA model, GAM model and BSTS model (Table1). We have observed that the BSTS model demonstrates superior performance in this study. The BSTS model outperforms the ARIMA model and GAM model in key performance metrics such as Root Mean Square Error (RMSE), Mean Absolute Percentage Error (MAPE), and R-squared (R2). Consequently, we have chosen to apply the BSTS model for our study.

**Table1** Comparison of fitting goodness between BSTS model, ARIMA model and GAM model

| Variables | Model prediction（2018-2019） | | |
| --- | --- | --- | --- |
|  | BSTS | ARIMA | GAM |
| RMSE | 1.14 | 1.17 | 1.15 |
| MAPE | 29.31% | 75.70% | 84.00% |
| R^2^ | 0.84 | 0.81 | 0.83 |

2. Thank you for your insights and for highlighting the importance of public health interventions for the mosquito vector. We have reconsidered the public health measures in the manuscript, and the revised measures are more practical and relevant, as follows:

“As a high-risk region of dengue epidemic, Guangdong Province continued to pay attention to and even strengthen dengue prevention and control during the 2020-2022 period of COVID-19 pandemic, particularly during the dengue epidemic season. For instance, to prevent the risk of potential dengue importation, Guangdong implemented stricter health surveillance and screening measures for inbound traveler from regions where dengue was endemic, such as enhanced screening for Dengue Virus NS1 antigen. Regular monitoring of mosquito vector density conducted to identify high-risk areas for vector breeding, facilitating timely vector control and breeding site sanitation. Based on the Joint Prevention and Control Mechanisms against on COVID-19 [2], Guangdong has strengthened collaboration among multiple departments, including the centers for disease control and prevention, hospitals, police, transportation, and urban management. If new local dengue cases are identified, these mechanisms ensure that the prevention and control responsibilities are firmly placed on local government, organizations, departments, and individuals. This has led to early case detection and vector control measures, facilitating the immediate control of local dengue outbreaks. Concurrently, health education was intensified to elevate public awareness of dengue prevention, and encourage the public to participate in the patriotic health action focusing on vector control and mosquito elimination, such as the removal of stagnant water and the reduction of mosquito breeding sites.”

3. We agree with you about the impact of COVID-19 interventions on mosquito vectors. Our research and available data indicate that during the COVID-19 pandemic, the mosquito vector density in Guangdong Province was lower in 2020-2022 than in 2017-2019 (Fig1). During the COVID-19 pandemic, increased public health awareness has led to increased emphasis on vector control measures, thereby has contributed to reduce mosquito vector breeding. We have added the related description of the impact of COVID-19 on mosquito vector-related interventions in the Materials and Methods section (Line170-173), please refer to the following content:

“Concurrently, health education was intensified to elevate public awareness of dengue prevention, and encourage the public to participate in the patriotic health action focusing on vector control and mosquito elimination, such as the removal of stagnant water and the reduction of mosquito breeding sites.”

Fig1 Temporal distribution of mosquito vector density (BI and MOI) in Guangdong Province from 2017 to 2022

**2. Academic Editor**

**From AE:** The study found a significant correlation between dengue incidence in Guangdong and Southeast Asian (SEA) countries, indicating that the dynamics of dengue transmission in the region are interlinked. But, what specific public health interventions were implemented in Guangdong Province during the COVID-19 pandemic that contributed to the significant decline in dengue cases from 2020 to 2022?

**Response:**

Thank you for your valuable comments on our study. The significantly decreased of dengue cases in Guangdong Province from 2020 to 2022 may due to COVlD-19 interventions and additional vector control measures. For imported dengue cases, Guangdong implemented stricter health surveillance and screening measures for inbound traveler from regions where dengue is endemic. Guangdong has implemented a 14-day centralized quarantine on inbound travelers. The majority of imported dengue cases are detected at ports of entry and during centralized isolation, effectively reducing the transmission risk of imported cases from Southeast Asian countries [3,4]. For local dengue cases, leveraging the Joint Prevention and Control Mechanisms established against COVID-19, immediate implementation of control measures such as dengue case management and vector elimination can be initiated. According to your comments, we have added the relative public health interventions for dengue during 2020-2022 in the Materials and Methods section (Line155-173), please find the follows:

“As a high-risk region of dengue epidemic, Guangdong Province continued to pay attention to and even strengthen dengue prevention and control during the 2020-2022 period of COVID-19 pandemic, particularly during the dengue epidemic season. For instance, to prevent the risk of potential dengue importation, Guangdong implemented stricter health surveillance and screening measures for inbound traveler from regions where dengue was endemic, such as enhanced screening for Dengue Virus NS1 antigen. Regular monitoring of mosquito vector density conducted to identify high-risk areas for vector breeding, facilitating timely vector control and breeding site sanitation. Based on the Joint Prevention and Control Mechanisms against on COVID-19 [2], Guangdong has strengthened collaboration among multiple departments, including the centers for disease control and prevention, hospitals, police, transportation, and urban management. If new local dengue cases are identified, these mechanisms ensure that the prevention and control responsibilities are firmly placed on local government, organizations, departments, and individuals. This has led to early case detection and vector control measures, facilitating the immediate control of local dengue outbreaks. Concurrently, health education was intensified to elevate public awareness of dengue prevention, and encourage the public to participate in the patriotic health action focusing on vector control and mosquito elimination, such as the removal of stagnant water and the reduction of mosquito breeding sites.”

**Reference:**

3.	Zhao L, Guo X, Li L, Jing Q, Ma J, Xie T, et al. Phylodynamics unveils invading and diffusing patterns of dengue virus serotype-1 in Guangdong, China from 1990 to 2019 under a global genotyping framework. Infectious diseases of poverty. 2024;13(1):43.

4.	Tan XH, Deng AP, Zhang YT, Luo M, Deng H, Yang YW, et al. Analysis of the impact of health management measures for entry personnel on imported Dengue fever in Guangdong Province, 2020-2022. Chin J Epidemiol. 2023;44(6):954-9.

**3. Reviewer #1:**
**3.1 Summary and General Comments**

Reviewer #1: The authors assessed the temporal shifts in dengue in Guangdong Province before and during the COVID-19 pandemic, using Bayesian structural time series (BSTS) models to forecast transmission trends without public health intervention measures or pandemic. The study found that the transmission patterns of dengue have been markedly reshaped during the COVID-19 pandemic. The findings further supported the notion that the dengue is not currently endemic in Guangdong. Overall, it is well-written and is a useful research contribution.
I have several comments for authors to consider.

**Comment 1: In the introduction, please add some information about the disease burden of dengue in Guangdong Province.**

**Response:**

We greatly appreciate your valuable suggestions. Incorporating information about the burden of dengue in Guangdong Province can help readers understand the necessity of this study. We have made corresponding additions in the introduction (Line 105-108), as follows:
 "Guangdong Province has been a region with a high incidence of dengue [5]. From 2005 to 2020, China reported 81,648 local cases of dengue, with Guangdong accounting for 74% of dengue cases in China [6]. In general, the dengue epidemic in Guangdong Province is driven by importation [7, 8]."

**Reference:**

5.	Li Y, Xu J, Zhong D, Zhang H, Yang W, Zhou G, et al. Evidence for multiple-insecticide resistance in urban Aedes albopictus populations in southern China. Parasites & vectors. 2018;11(1):4.

6.	YUE Y-j, LIU X-b, REN D-s, YIN W-w. Epidemiological characteristics of dengue fever in Chinese mainland 2005-2020. Chin J Vector Biol & Control. 2023;34(06):761-6+818.

7.	Shen SQ, Wei HX, Fu YH, Zhang H, Mo QY, Wang XJ, et al. Multiple Sources of Infection and Potential Endemic Characteristics of the Large Outbreak of Dengue in Guangdong in 2014. Scientific reports. 2015;5:16913.

8.	Lu J, Chen Z, Ma M, Cai R, Su W, Li Y, et al. Epidemic trend of dengue fever in Guangzhou city from 2008 to 2017. J Trop Med. 2018;18(07):973-6+85.

**Comment 2：Line 81-83, 390 million infections estimated by WHO should give the time when estimated.**

**Response:**

Thank you for your insightful comments. We agree with you that specific times can improve the clarity and credibility of our manuscripts. This estimation was conducted in 2013, we have added the relevant information in the introduction (Line 83-86):

“The estimates of dengue disease burden in 2013 suggested that 390 million dengue infections occur per year, resulting in 96  million symptomatic infections [9] and 20,000 deaths [10], with transmission occurring in at least 128 countries [11]. ”

**Reference：**

9.	Bhatt S, Gething PW, Brady OJ, Messina JP, Farlow AW, Moyes CL, et al. The global distribution and burden of dengue. Nature. 2013;496(7446):504-7.

10.	Stanaway JD, Shepard DS, Undurraga EA, Halasa YA, Coffeng LE, Brady OJ, et al. The global burden of dengue: an analysis from the Global Burden of Disease Study 2013. The Lancet Infectious diseases. 2016;16(6):712-23.

11.	Brady OJ, Gething PW, Bhatt S, Messina JP, Brownstein JS, Hoen AG, et al. Refining the global spatial limits of dengue virus transmission by evidence-based consensus. PLoS neglected tropical diseases. 2012;6(8):e1760.

**Comment 3：In the materials and methods, please add a description of the criteria for identifying "dengue cases"**

**Response:**

Thank you for your valuable feedback. Dengue cases were diagnosed according to the National Diagnostic Criteria for Dengue Fever (WS216-2008 and WS216-2018) [12]. We have supplemented these definitions in the manuscript to enhance understanding of our case identification process, as follows (Line 121-122):

“Dengue cases were diagnosed according to the National Diagnostic Criteria for Dengue Fever (WS216-2008 and WS216-2018). Imported dengue cases were defined as those that had been to a dengue-endemic country or region within 14 days before the onset.”

**Reference:**

12.	Jiang L, Liu Y, Su W, Liu W, Dong Z, Long Y, et al. Epidemiological and genomic analysis of dengue cases in Guangzhou, China, from 2010 to 2019. Scientific reports. 2023;13(1):2161.

**Comment 4 and Comment 5:**

4. Some teams used GAM or ARIMA model other than BSTS model to predict the incidence of dengue, have the authors tried this model on their data. If so, what is the difference between these two models in prediction？
5. As for this issue, I would like to see the details and the difference of these two models in this study. The author better provide the analysis of GAM or ARIMA model for evaluation and comparison.

**Response:**

Thank you for your valuable suggestions. Following your suggestions, we have conducted ARIMA model study and GAM model study. We subsequently compared the result from the ARIMA model, GAM model and BSTS model. Through comparative analysis, we have found that the BSTS model demonstrated superior performance in our study. The BSTS model outperformed the ARIMA model and GAM model in key performance indicators such as Root Mean Square Error (RMSE), Mean Absolute Percentage Error (MAPE), and R-squared (R^2^) (**Table1, Fig2**). Lower RMSE and MAPE values indicate better performance, while a higher R^2^ value indicates a better model fit. Additionally, compared to ARIMA, the BSTS model has a narrower confidence interval, thereby enhancing its superior fit. Based on our analysis, we have chosen to employ the BSTS model for our study.

**Table1** Comparison of fitting goodness between BSTS model, ARIMA model and GAM model

| Variables | Model prediction（2018-2019） | | |
| --- | --- | --- | --- |
|  | BSTS | ARIMA | GAM |
| RMSE | 1.14 | 1.17 | 1.15 |
| MAPE | 29.31% | 75.70% | 84.00% |
| R^2^ | 0.84 | 0.81 | 0.83 |

**Fig2** The comparison between models in fitting of dengue incidence in Guangdong Province during 2012-2019. (A) BSTS model. (B) ARIMA model. (C) GAM model.

**4. Reviewer #2:**

**4.1 Methods**

Reviewer #2: The authors aim to evaluate the impact of public health interventions on the incidence of dengue. However, the statistical methodologies used, such as the reduction in cases by comparing the forecast with the observed cases, do not allow for this level of causal inference. I suggest that the objectives be rewritten to make it clear that the study only evaluates the trend and temporal forecast of cases. In the discussion, I suggest that the authors address the possibility that part of this reduction is likely associated with the health interventions adopted.

**Response:**

Thank you for your thoughtful comments and for highlighting the importance of clarity in our study objectives and the limitations of our statistical methods in drawing causal inferences. We have revised the text accordingly.

1. Regarding the objectives of the study, we totally agree with your suggestions. We have revised the study objectives to more accurately reflect the scope and contribution of our study. The objectives are revised as follows:

“We aimed to analyze the epidemiological characteristics of dengue in Guangdong Province, China, and to investigate the temporal shifts in dengue epidemic in Guangdong Province during the COVID-19 pandemic.”

2. With regard to causal inference, we have made the relevant revisions into the manuscript. Firstly, the study objectives have been revised. Our study is not a rigorous causal inference, but rather aims to investigate the temporal shifts in dengue epidemic in Guangdong Province during the COVID-19 pandemic. Secondly, several studies have shown that COVID-19 interventions have a significant impact on dengue incidence. Studies have demonstrated that Nonpharmaceutical interventions (NPIs) implemented during the COVID-19 pandemic [12,13], such as strengthening quarantine of inbound travelers, restricting population movement, and maintaining social distancing, are effective in controlling dengue transmission. Thirdly, previous studies have shown that using time series models to investigate the co-benefits of COVID-19 interventions on the notifiable infectious diseases in China [13, 14], such as dengue and hand, foot and mouth disease, has a significant decreasing effect. These indicate that the method used in this study would be feasible. However, as a time-series analyses of ecological study design, the ability for causal inference is relative lower. Regarding this issue, we added a supplement to the limitation (Line 400-404).

“Third, this study is an ecological study design, and the direct effects of specific public health interventions on dengue need further investigation. In addition, dengue transmission may be influenced by complex factors, and although multiple factors were accounted for in the model construction process, there may still be some factors that were not considered.”

3. We have supplemented the relevant public health interventions in the method (Line 155-173) and discussion section (Line 355-372) , as follows:

“As a high-risk region of dengue epidemic, Guangdong Province continued to pay attention to and even strengthen dengue prevention and control during the 2020-2022 period of COVID-19 pandemic, particularly during the dengue epidemic season. For instance, to prevent the risk of potential dengue importation, Guangdong implemented stricter health surveillance and screening measures for inbound traveler from regions where dengue was endemic, such as enhanced screening for Dengue Virus NS1 antigen. Regular monitoring of mosquito vector density conducted to identify high-risk areas for vector breeding, facilitating timely vector control and breeding site sanitation. Based on the Joint Prevention and Control Mechanisms against on COVID-19 [2], Guangdong has strengthened collaboration among multiple departments, including the centers for disease control and prevention, hospitals, police, transportation, and urban management. If new local dengue cases are identified, these mechanisms ensure that the prevention and control responsibilities are firmly placed on local government, organizations, departments, and individuals. This has led to early case detection and vector control measures, facilitating the immediate control of local dengue outbreaks. Concurrently, health education was intensified to elevate public awareness of dengue prevention, and encourage the public to participate in the patriotic health action focusing on vector control and mosquito elimination, such as the removal of stagnant water and the reduction of mosquito breeding sites.”

“From 2020 to 2022, the dengue incidence in Guangdong Province decreased significantly. There may be several reasons for this. Firstly, during the COVID-19 pandemic, the implementation of entry restrictions and quarantine measures in Guangdong led to the detection of the vast majority of imported dengue cases at entry ports and during centralized isolation, effectively reducing the risk of dengue importation. Secondly, targeted quarantine and surveillance of inbound travelers from dengue epidemic regions facilitated the timely identification of dengue cases, which likely resulted in a large reduction in transmission. Thirdly, for local dengue cases, the Joint Prevention and Control Mechanisms established during COVID-19 enabled the immediate implementation of control measures for infectious disease [2], such as dengue case management and vector control. These actions effectively reduced the risk of community transmission of dengue. Additionally, the heightened public health awareness led to greater emphasis on proactive prevention and prompt medical consultation, which is beneficial for the early detection, diagnosis, and treatment of cases, and further reduces the transmission of dengue.”

**Reference:**

12.	Surendran SN, Nagulan R, Sivabalakrishnan K, Arthiyan S, Tharsan A, Jayadas TTP, et al. Reduced dengue incidence during the COVID-19 movement restrictions in Sri Lanka from March 2020 to April 2021. BMC public health. 2022;22(1):388.

13.	Zheng D, Shen L, Wen W, Ling F, Miao Z, Sun J, et al. The impact of EV71 vaccination program on hand, foot and mouth disease in Zhejiang Province, China: A negative control study. Infectious Disease Modelling. 2023;8(4):1088-96.

14.	Li K, Rui J, Song W, Luo L, Zhao Y, Qu H, et al. Temporal shifts in 24 notifiable infectious diseases in China before and during the COVID-19 pandemic. Nature communications. 2024;15(1):3891.

**4.2 Results**

Reviewer #2: As stated above, I believe that the reduction in expected cases based on the observed cases does not allow for the causal inference that the authors aim for in this study, that is, to claim that a 99% reduction in dengue cases is due to the impacts of health interventions adopted since 2020.

**Response:**

Thank you for your insightful comments. The causal inference issue has been replied to in the above comments. Concerning the statements of results, we have been more cautious in our manuscript, avoiding direct claims of causal inference in the result section. The result of the model study was revised in the abstract section as follows.

“According to BSTS model estimates, 6557 local dengue cases may have been reduced in Guangdong Province from 2020 to 2022, with a relative reduction of 99.91% (95%CI: 98.85－99.99%). ”

**4.3 Conclusions**

Reviewer #2: As stated above, I believe that the reduction in expected cases based on the observed cases does not allow for the causal inference that the authors aim for in this study, that is, to claim that a 99% reduction in dengue cases is due to the impacts of health interventions adopted since 2020.

**Response:**

Thanks for your valuable comments. As replied in the first comments, the time series model analysis in this study was not a classic causal inference study design. We have made the relevant revisions to the whole manuscript.

**4.4 Summary and General Comments**
In this study, the authors suggest that the more than 99% reduction in local dengue cases in the studied region was due to public health interventions. They indicate that measures such as restrictions on people’s movement and gatherings, as well as increased health surveillance (lines 344-347), were effective in reducing dengue cases. Additionally, the authors suggest that "restricting international flights, strengthening entry management, and quarantining immigration from dengue epidemic regions can reduce the occurrence of dengue" (lines 347-350). However, over 96% of the dengue cases in the studied region are local, not imported, indicating that drastic measures like air traffic control and immigration quarantines may have some effect, but this effect is likely to be limited, as the vast majority of cases in the studied region were autochthonous. In other words, the authors need to consider this, as the way it was presented suggests that the primary responsibility for the cases in the region lies with the movement of people from outside of China, when in fact it is the opposite.

**Response:**

Thanks for your valuable suggestions. In Guangdong, dengue is not endemic, the local dengue epidemic was mainly triggered by imported cases. During the COVID-19 pandemic, the prevention and control of dengue also implemented the strategy of "preventing external importation and internal infection." During this period, Guangdong intensified quarantine, testing, and health management for individuals from dengue-endemic regions. Additionally, during the peak dengue transmission season from May to October, based on the Joint Prevention and Control Mechanisms against COVID-19, Guangdong enhanced cooperation among multiple departments such as centers for disease control and prevention, hospitals, police, transportation, and urban management. This collaborative effort emphasized the responsibilities of "local government, organizations, departments, and individuals," which facilitated the detection and control of new local dengue cases. Consequently, from 2020 to 2022, there were only six local dengue cases reported, with no large-scale local dengue outbreaks occurring. We fully agree with and appreciate your suggestions regarding the measures taken for local dengue outbreaks. We have further elaborated on this content in the methods and discussion sections. For details, please refer to the revised and supplemented sections on Methods and discussion.

“As a high-risk region of dengue epidemic, Guangdong Province continued to pay attention to and even strengthen dengue prevention and control during the 2020-2022 period of COVID-19 pandemic, particularly during the dengue epidemic season. For instance, to prevent the risk of potential dengue importation, Guangdong implemented stricter health surveillance and screening measures for inbound traveler from regions where dengue was endemic, such as enhanced screening for Dengue Virus NS1 antigen. Regular monitoring of mosquito vector density conducted to identify high-risk areas for vector breeding, facilitating timely vector control and breeding site sanitation. Based on the Joint Prevention and Control Mechanisms against on COVID-19 [2], Guangdong has strengthened collaboration among multiple departments, including the centers for disease control and prevention, hospitals, police, transportation, and urban management. If new local dengue cases are identified, these mechanisms ensure that the prevention and control responsibilities are firmly placed on local government, organizations, departments, and individuals. This has led to early case detection and vector control measures, facilitating the immediate control of local dengue outbreaks. Concurrently, health education was intensified to elevate public awareness of dengue prevention, and encourage the public to participate in the patriotic health action focusing on vector control and mosquito elimination, such as the removal of stagnant water and the reduction of mosquito breeding sites.”

“From 2020 to 2022, the dengue incidence in Guangdong Province decreased significantly. There may be several reasons for this. Firstly, during the COVID-19 pandemic, the implementation of entry restrictions and quarantine measures in Guangdong led to the detection of the vast majority of imported dengue cases at entry ports and during centralized isolation, effectively reducing the risk of dengue importation. Secondly, targeted quarantine and surveillance of inbound travelers from dengue epidemic regions facilitated the timely identification of dengue cases, which likely resulted in a large reduction in transmission. Thirdly, for local dengue cases, the Joint Prevention and Control Mechanisms established during the COVID-19 enabled the immediate implementation of control measures for infectious disease, such as dengue case management and vector control. These actions effectively reduced the risk of community transmission of dengue. Additionally, the heightened public health awareness led to greater emphasis on proactive prevention and prompt medical consultation, which is beneficial for the early detection, diagnosis, and treatment of cases, and further reduce the transmission of dengue. Overall, the decline in dengue cases in Guangdong during the COVID-19 pandemic was related to the strengthening of quarantine measures for imported dengue cases and the early detection and intervention of local dengue cases.”

Moreover, public health interventions are important in controlling dengue; however, it is neither expected nor feasible, from a public health and surveillance standpoint, to achieve a 99% reduction in the incidence of a vector-borne disease, carried by a winged insect highly adapted to the urban environment, merely through measures like controlling movement and gatherings. It cannot be that easy to control dengue, as the study seems to imply. Dengue, like most neglected vector-borne diseases, is much more closely associated with social factors than with a lack of surveillance and health interventions. In other words, it is far more associated with poverty, social inequality, and other chronic structural problems in the societies where it occurs endemically or epidemically. Therefore, it is not plausible to believe that a 99% reduction in incidence occurred because the government controlled people’s movement or something similar. If that were the case, countries like Brazil, which implement important surveillance and health intervention measures such as early diagnosis, immediate treatment, spraying, and health education, would have already achieved some level of success in controlling this disease. However, the opposite is observed, as dengue incidence continues to rise worldwide, even spreading to previously unaffected regions such as Europe. It is also worth noting again that the vector is highly adapted to urban environments and is winged, so while movement restrictions may have some impact, their effect on dengue control is notoriously limited.
**Response:**

1. Thank you for your valuable suggestions. This result is plausible with a 99% reduction in dengue cases in Guangdong Province during the COVID-19 pandemic. This significant effect is closely related to the characteristics of dengue epidemic in Guangdong Province. Dengue epidemic in Guangdong Province is driven by importation and that dengue is not currently endemic. In Guangdong, the prevention and control of dengue implemented the strategy of "preventing external importation and internal infection.

To prevent the risk of potential dengue importation, Guangdong implemented stricter health surveillance and screening measures for inbound travelers from regions where dengue is endemic. During the COVID-19 pandemic, Guangdong Province has implemented a 14-day centralized quarantine on all inbound travelers. The majority of imported dengue cases are detected at ports of entry and during centralized isolation, effectively reducing the transmission risk of imported cases [4].

For the prevention of local dengue transmission, the Joint Prevention and Control Mechanisms established during the COVID-19 pandemic enabled the immediate implementation of control measures, such as case management and vector control. These actions effectively reduced the risk of community transmission of dengue.

On the whole, we agree with you that dengue is an exceedingly challenging vector-borne disease to control. During the COVID-19 pandemic, the stringent public health interventions and the establishment of special prevention and control mechanisms in Guangdong have made it possible to effectively manage this disease locally.

Thus, I strongly believe that the more than 99% reduction in local cases since 2020 is due to underreporting, caused by several factors such as low demand for diagnosis and a decreased sensitivity of the local surveillance system in diagnosing and treating potential cases due to the overwhelming burden of COVID-19. This happened globally with various diseases.

**Response:**

Thank you for concerning the issue of potential underreporting of dengue cases. During the COVID-19 pandemic, the likelihood of dengue underreporting in Guangdong Province is minimal. The reasons are as follows:

**Firstly**, from January 2020 to November 2022, China implemented a 'dynamic zero-COVID' strategy, which effectively controlled the COVID-19 outbreaks. The incidence of COVID-19 in Guangdong was 12.6/100,000 population (15,989 cases) from January 2020 to June 2022 [1]. During this period, the healthcare system in Guangdong Province continued to function normally, allowing dengue cases to be treated promptly and thus reducing the likelihood of underreporting cases of dengue. **Secondly**, during 2020-2022, Guangdong implemented stricter health surveillance for fever symptoms, which are common in both COVID-19 and dengue. A person with fever symptom was promptly treated, this enhanced surveillance likely heightened the detection of dengue cases. Hospitals maintained sufficient sensitivity in diagnosing dengue cases, such as conducting NS1 antigen testing for suspected dengue virus cases in secondary and higher-level medical institutions across the province. Contrary to surveillance sensitivity before 2020, the monitoring and diagnosis of dengue cases have been enhanced during the COVID-19 pandemic. **Thirdly**, regarding dengue case reports, China classifies dengue as a Category B notifiable disease, requiring reporting within 24 hours of diagnosis. During the COVID-19 pandemic, Guangdong has consistently organized annual surveys and evaluations of the quality and management of notifiable infectious disease reporting across all levels of medical and disease control institutions in every city. The surveys indicated an average infectious disease reporting rate of 95%. Notably, the port cities of Guangzhou, Shenzhen, and Zhuhai have achieved reporting rates exceeding 99%, suggesting a low likelihood of underreporting for dengue. Nevertheless, there is still the possibility of underreporting, and we have added relevant descriptions in the limitations (Line 392-394):

“First, some dengue cases may have been under-diagnosed and under-reported to some extent from 2020 to 2022, due to the burden of COVID-19 control and prevention.”

In other words, it is important to discuss in more detail that most of the cases in the studied region were local, so suggesting that drastic measures such as air traffic and immigration control, as proposed by the authors as strategies for improving dengue control, need to be reconsidered. Furthermore, the authors need to emphasize that the study contributes by descriptively analyzing the drop in incidence since 2020, but it lacks the causal and epidemiological basis to assert that the causes of this drastic drop were the government’s COVID-related public health intervention measures, such as traffic control and restrictions on gatherings. As I mentioned above, I do not believe it would be that easy to control dengue.

**Response:**

Thank you for your thorough review and constructive feedback on our manuscript. We responded as follows:

1. We agree with you that prevention and control measures for local cases are important. We have added public health interventions for local cases to the discussion. The additional public health interventions are as follows:

“As a high-risk region of dengue epidemic, Guangdong Province continued to pay attention to and even strengthen dengue prevention and control during the 2020-2022 period of COVID-19 pandemic, particularly during the dengue epidemic season. For instance, to prevent the risk of potential dengue importation, Guangdong implemented stricter health surveillance and screening measures for inbound traveler from regions where dengue was endemic, such as enhanced screening for Dengue Virus NS1 antigen. Regular monitoring of mosquito vector density conducted to identify high-risk areas for vector breeding, facilitating timely vector control and breeding site sanitation. Based on the Joint Prevention and Control Mechanisms against on COVID-19 [2], Guangdong has strengthened collaboration among multiple departments, including the centers for disease control and prevention, hospitals, police, transportation, and urban management. If new local dengue cases are identified, these mechanisms ensure that the prevention and control responsibilities are firmly placed on local government, organizations, departments, and individuals. This has led to early case detection and vector control measures, facilitating the immediate control of local dengue outbreaks. Concurrently, health education was intensified to elevate public awareness of dengue prevention, and encourage the public to participate in the patriotic health action focusing on vector control and mosquito elimination, such as the removal of stagnant water and the reduction of mosquito breeding sites.”

“From 2020 to 2022, the dengue incidence in Guangdong Province decreased significantly. There may be several reasons for this. Firstly, during the COVID-19 pandemic, the implementation of entry restrictions and quarantine measures in Guangdong led to the detection of the vast majority of imported dengue cases at entry ports and during centralized isolation, effectively reducing the risk of dengue importation. Secondly, targeted quarantine and surveillance of inbound travelers from dengue epidemic regions facilitated the timely identification of dengue cases, which likely resulted in a large reduction in transmission. Thirdly, for local dengue cases, the Joint Prevention and Control Mechanisms established during the COVID-19 enabled the immediate implementation of control measures for infectious disease, such as dengue case management and vector control. These actions effectively reduced the risk of community transmission of dengue. Additionally, the heightened public health awareness led to greater emphasis on proactive prevention and prompt medical consultation, which is beneficial for the early detection, diagnosis, and treatment of cases, and further reduces the transmission of dengue. Overall, the decline in dengue cases in Guangdong during the COVID-19 pandemic was related to the strengthening of quarantine measures for imported dengue cases and the early detection and intervention of local dengue cases.”

2. Thanks for your valuable suggestions. The causal inference has been answered in the Methods and the Results section.
Finally, We would appreciate your careful comments and valuable suggestions again.
With your suggestions, we have made many revisions and improvements, especially supplementing the prevention and control measures of dengue during the COVID-19 and the response to local dengue outbreaks. We believe that this manuscript has been greatly improved. Thank you!
